# Population spikes in cortical networks during different functional states

**DOI:** 10.3389/fncom.2012.00043

**Published:** 2012-07-13

**Authors:** Shirley Mark, Misha Tsodyks

**Affiliations:** Department of Neurobiology, Weizmann Institute of ScienceRehovot, Israel

**Keywords:** neural network, synchrony and synaptic depression

## Abstract

Brain computational challenges vary between behavioral states. Engaged animals react according to incoming sensory information, while in relaxed and sleeping states consolidation of the learned information is believed to take place. Different states are characterized by different forms of cortical activity. We study a possible neuronal mechanism for generating these diverse dynamics and suggest their possible functional significance. Previous studies demonstrated that brief synchronized increase in a neural firing [Population Spikes (PS)] can be generated in homogenous recurrent neural networks with short-term synaptic depression (STD). Here we consider more realistic networks with clustered architecture. We show that the level of synchronization in neural activity can be controlled smoothly by network parameters. The network shifts from asynchronous activity to a regime in which clusters synchronized separately, then, the synchronization between the clusters increases gradually to fully synchronized state. We examine the effects of different synchrony levels on the transmission of information by the network. We find that the regime of intermediate synchronization is preferential for the flow of information between sparsely connected areas. Based on these results, we suggest that the regime of intermediate synchronization corresponds to engaged behavioral state of the animal, while global synchronization is exhibited during relaxed and sleeping states.

## Introduction

Cortical activity was shown to depend critically on behavioral state of the animal. Experiments reveal that different frequency ranges are dominant in slow wave sleep (SWS), rapid eye movement sleep (REM) and different wake states (Steriade et al., 1993; Harris and Thiele, 2011). Various cortical states can also be seen in recording of membrane potential in awake animals (Poulet and Petersen, 2008; Okun et al., 2010). The influence of behavioral states on network dynamics is observed throughout the cortex, beginning from the primary sensory cortices (Harris and Thiele, 2011).

Recording extracellular activity from the somatosensory cortex (S1) and auditory cortex of rats showed that response to a stimulus is larger in the passive states compare to the active state (Fanselow and Nicolelis, 1999; Castro-Alamancos, 2004; Otazu et al., 2009). Intracellular recordings from S1 of mice revealed larger fluctuations in the membrane potential and larger correlations between neighboring neurons during quiet wake state compared to whisking state, while the mean firing rate of pyramidal neurons did not change significantly between these states. It appears that changes in neural dynamics originate from internal regulation because sensory inputs have no significant effect on global properties of neural dynamics at all behavioral states (Poulet and Petersen, 2008; Gentet et al., 2010).

There are various models for generating network activity synchronization (Sturm and Konig, 2001). In a recurrent network model with short-term synaptic depression (STD) there is a parameter regime in which short synchronized bursts of activity [Population Spike (PS)] can emerge spontaneously at a low frequency (Tsodyks et al., 2000; Loebel and Tsodyks, 2002). This type of synchronized events was confirmed experimentally in the auditory cortex (DeWeese and Zador, 2006). In this study we considered clustered networks divided into strongly interconnected groups of neurons. This clustered architecture was inspired by experimental studies on cortical connectivity (Song et al., 2005; Yoshimura et al., 2005).

It was previously proposed that network synchronization can ensure propagation of signals from one area to another in the sparsely connected cortex (Singer, 1993); thus controlling network synchronization may have an important functional role. Transitions between different behavioral and neural states can be accomplished by activation of different neuromodulatory systems (Steriade et al., 1993). These systems influence all of the forebrain in a diffusive way (Hasselmo, 1995) and can alter network dynamics via their effect on neurons and synaptic connections (Steriade et al., 1993; Marder and Thirumalai, 2002; Giocomo and Hasselmo, 2007). Synaptic depression can be regulated by neuromodulators that change the release probability in intracortical connections (Tsodyks and Markram, 1997; Wu and Saggau, 1997). Consequently, the emergence of PSs and their synchronization across strongly interconnected groups can be regulated, which results in controlling the flow of sensory information to distinct cortical areas.

Here we present a model which suggests a clear mechanism to control the level of synchronization in network activity. We show that the synchronization of noisy clustered network with STD can be shift smoothly from asynchronous to synchronous state by adjusting the release probability in recurrent connections. Synchronized activity can overcome the sparse connectivity between cortical areas; as a consequence, the flow of information from one cortical area to another can also be controlled.

## Methods

### Modeling cortical column

We represent a cortical column by a network of interconnected clusters; each one is divided into two units representing highly connected groups of excitatory and inhibitory neurons, respectively. Connections between units of different clusters are weaker then connections within clusters.

We used the rate model to describe the dynamics (Wilson and Cowan, 1972):

(1) $$\tau_{E}\frac{dE_{i}}{dt}=-E_{i}+\left(1-\tau_{\text{ref}}E_{i}\right){\left[\sum_{j}^{N}J_{ij}^{EE}\cdot \mathrm{Pr}\cdot x_{j}E_{j}+\sum_{j}^{N}J_{ij}^{EI}I_{j}+s\left(t\right)+\eta_{i}\left(t\right)+e_{E}\right]}^{+}$$

(2) $$\tau_{I}\frac{dI_{i}}{dt}=-I_{i}+{\left[\sum_{j}^{N}J_{ij}^{II}I_{j}+\sum_{j}^{N}J_{ij}^{IE}E_{j}+e_{I}\right]}^{+}$$

The *E*_*i*_ (*I*_*i*_) are the excitatory (inhibitory) rate variables for a corresponding unit in cluster *i.* τ_*E*_ (τ_*I*_) is the corresponding time constant. *N* is the number of clusters in the network. τ_ref_ determines the neurons' refractory period. Every unit receives synaptic inputs from all other units with synaptic efficacies *J*_αβ_ (pre-synaptic α neuron projects to post-synaptic β neuron, α; β = *E*; *I*). *e*_*E*_ (*e*_*I*_) is the mean background input, representing inputs from other brain areas or alternatively can represent mean resting membrane potential relative to threshold. *s(t)* is the external sensory input which is taken to be zero for spontaneous activity and otherwise as pulses with a certain duration (δ_*s*_) and amplitude (*A*_*s*_) that occur as random refractory Poisson process with a constant rate of $\frac{2}{3}$ Hz (the minimal time interval between pulses is 1 s). For simplicity we chose threshold —linear form of the neuronal gain function [*z*]^+^ = max (*z*, 0). We further introduced fluctuations to the input, η*(t)*, which is a time correlated noise with time constant τ_*n*_ and a standard deviation of $\frac{A_{n}}{\sqrt{2\tau_{n}}}$:

(3) $$\tau_{n}\frac{d\eta_{i}}{dt}=-\eta_{i}+A_{n}\xi \left(t\right)$$

We introduced synaptic depression in the excitatory-to-excitatory connections following a previous modeling work (Tsodyks et al., 1998). These synaptic connections are scaled by a factor (*Pr* · *x*_j_), where *x*_*j*_ is the average available synaptic resources in a unit *j* which decreases with unit activity and recovers to one with a time constant τ _*d*_; *Pr* is the release probability (same for all connections) and therefore the average fraction of synaptic resources that is utilized after each spike. The dynamics of the average available synaptic resources is governed by the following equation:

(4) $$\frac{dx_{i}}{dt}=\frac{1-x_{i}}{\tau_{d}}-\mathrm{Pr}\cdot x_{i}\cdot E_{i}$$

The parameters used in the simulations are listed in Table 1. The synaptic efficacies were adjusted such that the mean synaptic input from all other clusters is *A* time smaller then the mean inputs from within the cluster (see Table 1). We implemented the transitions between behavioral states by changing *Pr* in the range of 0.2–0.9. In order to keep the firing rate constant (2Hz), the external input *e*_*E*_ was adapted according to an empirical relationship (Figure 2C).

**Table 1:** **Rate model parameters**.

*N*	20	*A*_*n*_	0.15 s^−0.5^
τ_*E*_	10 ms	δ _*s*_	50 ms
τ_*I*_	10 ms	*A*_*s*_	1.5 Hz
τ_ref_	5 ms	*J*_*EE*_	5.25
τ_*d*_	400 ms	*J*_*IE*_	2.5
τ_*n*_	100 ms	*J*_*EI*_	−2.5
*e*_*I*_	0	*J*_*II*_	−8
**SYNAPTIC EFFICACIES IN/BETWEEN CLUSTERS**			
Diagonal element		$\frac{A}{A+1}J_{\alpha \beta }$	
Non-Diagonal elements		$\frac{J_{\alpha \beta }}{\left(A+1\right)\left(N-1\right)}$	

### Modeling readout population

The activity of the readout population is controlled by the following equation:

(5) $$\tau_{R}\frac{dR}{dt}=-R+J_{R}\sum_{i,j}^{N_{n},N}\sum_{\text{sp}}^{S_{i,j}}\delta \left(t-t_{\text{sp}}\right)$$

*R* is the firing rate variable, τ_*R*_ is its corresponding time constant and *J*_*R*_ is the synaptic efficacy of the readout synapses. *t*_sp_ is the spike time, *S*_*i,j*_ is the number of spikes emitted by neuron *i* belonging to a unit *j*. *N*_*n*_ is the number of excitatory neurons from each cluster that are connected to the readout. We chose τ_*R*_ = 10 ms and *J*_*R*_ = *1/N*. In order to study the effect of sparse connectivity, we changed the number of feed-forward neurons belonging to each unit that are connected to the readout (*N*_*n*_). The spike trains of neurons from a unit *j* were constructed as Poisson processes with a rate *E*_*j*_.

### Readout performance

We quantified the readout population performance by defining a threshold for detecting network activity events. We adopt terms from the receiver operating characteristic (ROC) nomenclature; True positive (TP) is a detected event that follows a stimulus and false positive (FP) is a spontaneous event. False negative (FN) refers to the situation when there was a stimulus but the readout population activity did not cross the threshold (Dayan and Abbott, 2001). We defined a time window for the network response to a stimulus by computing the per-istimulus time histogram (PSTH) of the readout population. The response period was taken to be the time duration after a stimulus in which the PSTH is above the mean value before stimulus (spontaneous activity). True negative event refers to a situation in which there was no stimulus and the activity did not cross the threshold. We simulated continuous activity, and consequently, almost the entire range of the simulation can account as True negative events. Taking these events into account in the readout performance will mask all other events. As a result, standard ROC analysis is not appropriate measure for our network performance. We therefore define a true positive ration (TPR) as a measure of performance:

$$TPR=\frac{TP}{TP+FN+FP}$$

We quantified the ability of the network to signal the occurrence of the stimulus by calculating the maximal *TPR* (*TPR*_max_) with respect to readout population detection threshold, for each values of *Pr* and *N*_*n*_ (we assume that a readout neuron can learn the optimal threshold, therefore it is not an important parameter in our examination).

### Synchrony measure

We calculated the global synchrony in the network as a normalized standard deviation of network firing rate, following a previous work (Golomb and Hansel, 2000):

$$\begin{array}{c} \text{syn}=\sqrt{\frac{\sigma_{E}^{2}}{\frac{1}{N_{e}}\sum_{i}^{N_{e}}\sigma_{E_{i}}^{2}}}\\ \sigma_{E}^{2}={\langle E{\left(t\right)}^{2}\rangle }_{t}-{\langle E\left(t\right)\rangle }_{t}^{2}\\ E\left(t\right)=\frac{1}{N_{e}}\sum_{i}^{N_{e}}E_{i}\left(t\right)\\ \sigma_{E_{i}}^{2}={\langle E_{i}{\left(t\right)}^{2}\rangle }_{t}-{\langle E_{i}\left(t\right)\rangle }_{t}^{2} \end{array}$$

*E*_*i*_ corresponds to firing rate of one unit (or neuron in the Integrate-and-Fire (I&F) network). The firing rate of a neuron in the I&F network was calculated with sliding window of 50 ms.

This synchrony measure is between 0 and 1, with 0 for asynchronous activity and 1 for fully synchronized activity.

### Integrate and fire network

Neurons were modeled as current based leaky integrate and fire units (Dayan and Abbott, 2001). The voltage membrane potential evolved according to the following equation:

(6) $$\tau_{m}=\frac{dV_{i}}{dt}=V_{0}-V_{i}+R_{in}[I_{\text{syn},i}+\eta_{i}(t)+A\xi_{i}(t)+F_{in}(t)]$$

where τ_*m*_ denotes the membrane time constant of a neuron, *V*_0_ is the neuron resting potential, *I*_syn_ is the recurrent synaptic current, η_*i*_*(t)* represents a non-specific background current (to excitatory neurons only) which was modeled as a time correlated noise, the same current for every neuron at the same unit [see Equation (3)] and ξ_*i*_*(t)* represents a non-specific background current which was modeled as a Gaussian white noise (different noise to each neuron). In the following, we incorporated the input resistance of the neuron, *R*_in_, into the currents, which were therefore measured in units of voltage (millivolts). Each time the membrane potential of a neuron reached threshold (–40 mv), a spike was emitted; then the neuron voltage was set to threshold voltage for 3 ms.

The synaptic current, *I*_syn_, was modeled as a summation of post-synaptic currents (PSCs) from all the pre-synaptic neurons connected to neuron (*i*). The excitatory-to-excitatory connections exhibit STD, therefore the synaptic current to an excitatory neuron follows the equation:

(7) $$\frac{dI_{\text{syn}}^{i}}{dt}=-\frac{I_{\text{syn}}^{i}}{\tau_{I}}+\sum_{\text{sp}}\sum_{j}^{N_{e}}A_{ij}\cdot {\mathrm{Pr}}_{j}\cdot x_{j}\delta_{j}\left(t-t_{j}^{\text{sp}}\right)+\sum_{\text{sp}}\sum_{j}^{N_{i}}A_{ij}\delta_{j}\left(t-t_{j}^{\text{sp}}\right)$$

The synaptic current to an inhibitory neuron is controlled by the following equation:

(8) $$\frac{dI_{\text{syn}}^{i}}{dt}=-\frac{I_{\text{syn}}^{i}}{\tau_{I}}+\sum_{\text{sp}}\sum_{j}^{N_{e}+N_{i}}A_{ij}\delta_{j}\left(t-t_{j}^{\text{sp}}\right)$$

τ_*I*_ is the synaptic current time constant, *N*_*e*_ and *N*_*i*_ are the number of excitatory and inhibitory neurons, respectively (*N*_*e*_ = 2000, *N*_*i*_ = 500).

The available synaptic resources (*x*_*i*_) decrease with every spike and recover with a time constant (τ_rec_):

(9) $$\frac{dx_{i}}{dt}=\frac{1-x_{i}}{\tau_{rec}}-{\mathrm{Pr}}_{i}x_{i}\sum_{\text{sp}}\delta \left(t-t_{i}^{\text{sp}}\right)$$

The membrane resting potential and the synaptic parameters (τ_rec_, *Pr*) were Gaussian distributed across the neurons with mean and variance given in Table 2. As before, we implemented the transitions between behavioral states by changing <*Pr*> in the range of 0.2–0.8. The membrane resting potential of the excitatory cells was adjusted such that the mean firing rate of the excitatory neurons was ∼2 Hz (the mean firing rate of the inhibitory neurons varied between 0.5 Hz and 1 Hz).

**Table 2:** **Parameters of the Integrate and Fire networks**.

	**Mean**	**Variance**						
T_*m*_	20 ms	—						
*A*_*n*_	$10\left[mV\sqrt{ms}\right],$	—						
τ_*n*_	10 ms	—						
*A*	10 mV	—						
*A*_*EE*_	4.43 mV	—						
*A*_*EI*_	–2.215 mV	—						
*A*_*IE*_	1.1 mV	—						
*A*_*II*_	–6.64 mV	—						
*Pr*	0.2–0.9	Mean/10						
τ_*rec*_	0.4 s	Mean/10						
*V*_0_	(–42)–(–48) mv	1.5 mv						
τ_*I*_	3 ms	—						
*P*_1_	0.165	—						
*P*_2_	0.022	—						
<*V*_0_>	−45.375	−45.875	−46.525	−47.2	−47.8	−48.28	−48.5	−48.8
<*Pr*>	0.1	0.2	0.3	0.4	0.5	0.6	0.7	0.8

The clustered architecture were constructed by assigning different connection probabilities between pairs of neurons belonging to the same cluster (*p*_1_) compared to different clusters (*p*_2_) and different synaptic efficacies (the synaptic efficacies were five time larger in the connections within cluster than between clusters) such that the ratio between the mean synaptic inputs from within the cluster and from other clusters is p1 × 5/[*p*2 × (*N* − 1)] ≈ 2 (*N* is the number of groups, *N* = 20).

The stimulus was simulated as pulses of a constant current (*F*_in_ = 1.5 mV, duration of 50 ms) that occur as random refractory Poisson process with a constant rate of $\frac{2}{3}$ Hz (the minimal time interval between pulses is 1 s).

## Results

**Figure 1:**
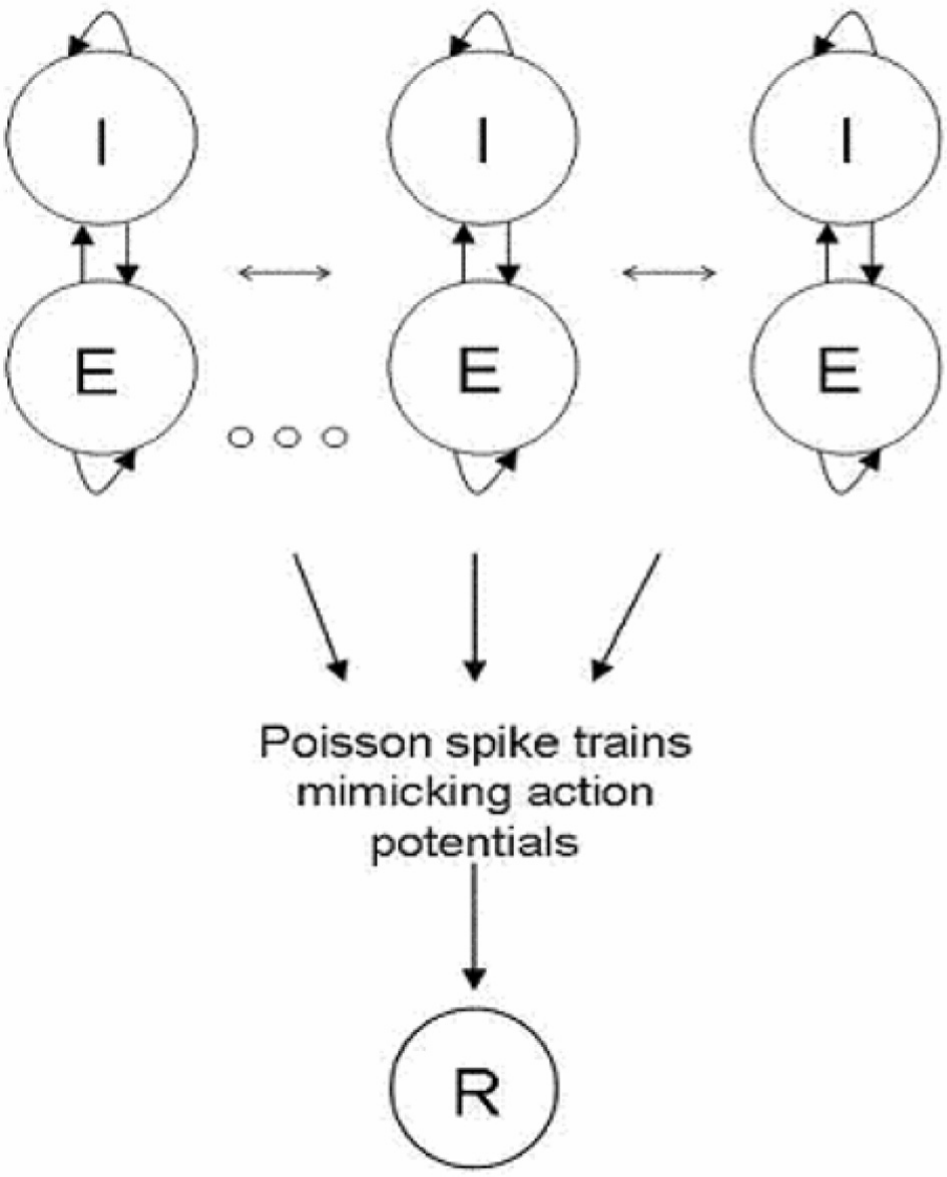
**Network Architecture.** Network is composed of clusters; each one is divided into two units representing highly connected groups of excitatory and inhibitory neural populations. All clusters are connected to each other; the connections within clusters are stronger than the connections between clusters. Excitatory neurons project feed—forward connections to a readout population (R). The input to the readout population is a summation over spike trains. The spike train of neurons from a certain unit was constructed as a Poisson process with the corresponding rate.

Inspired by experimental studies of cortical connectivity (Song et al., 2005; Yoshimura et al., 2005), we modeled a cortical column as a clustered recurrent network and explored its spontaneous dynamics and response to sensory stimulations. The network is composed of several clusters, each one divided into two units representing highly connected groups of excitatory and inhibitory neurons, respectively. Connections between units of different clusters are weaker then connections within clusters (see Figure 1). We used rate equations for the network dynamics such that each unit is described by one variable representing its average firing rate (See “Methods”). The excitatory-to-excitatory connections were endowed with activity dependent STD caused by depletion of synaptic resources. A fraction of available synaptic resources is utilized in response to an action potential and then recovers with the corresponding time constant (Tsodyks et al., 1998). In biological terms, this fraction reflects synaptic release probability (*Pr*). In addition to sensory input each excitatory unit receives random time-correlated noise current. (See “Methods” for details of the model). The ability of the network to transfer information about the occurrence of the stimulus was explored by quantifying the response of a readout population to changes in network activity following stimuli.

### Partial synchronization in clustered networks

**Figure 2:**
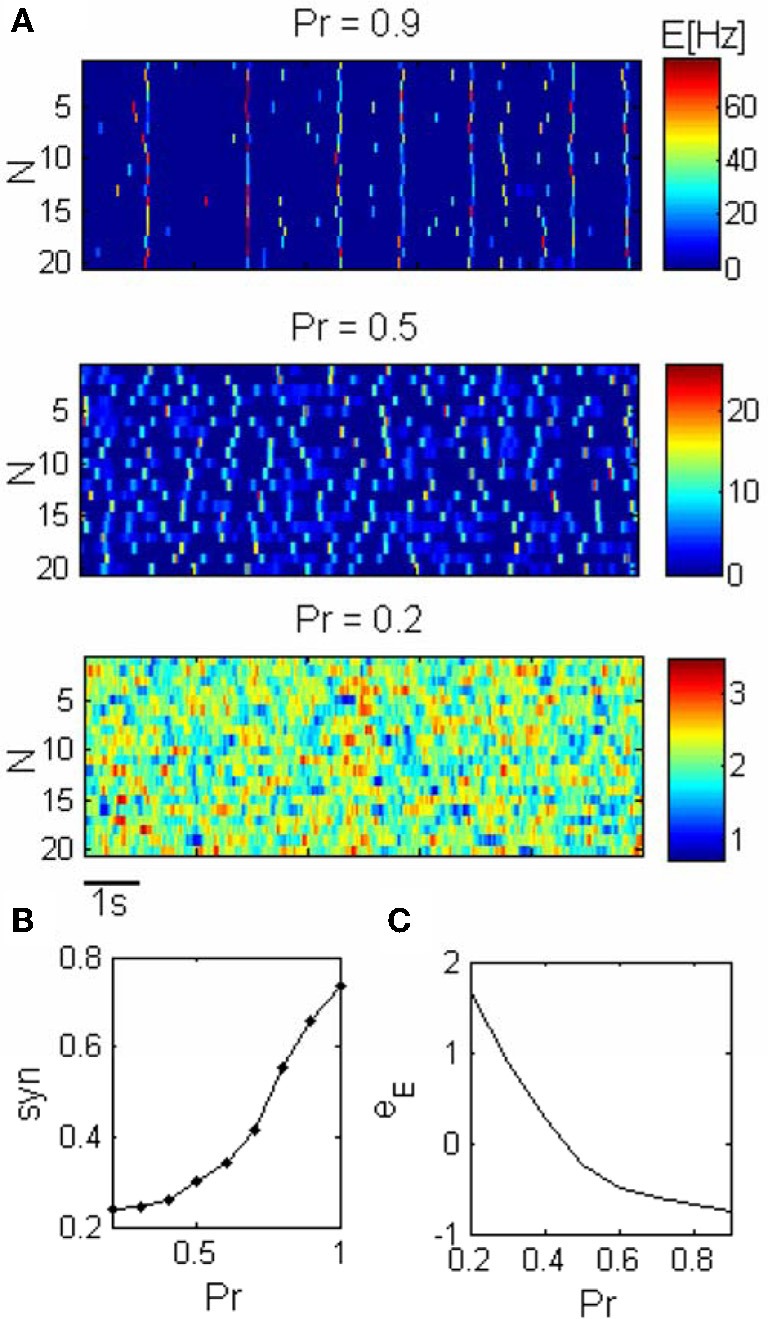
**Network dynamics across different states. (A)** Spontaneous activity of the excitatory units (*N* is the unit label). The synchronization in the network increases as a result of the increase in the release probability (*Pr*). *Pr* = 0.2: asynchronous activity, *Pr* = 0.5: PSs can occur within units but they are not synchronized. *Pr* = 0.9: Synchronous activity, PSs occur simultaneously in different clusters. **(B)** The synchronization grows smoothly with the release probability. **(C)** The mean resting potential was adapted according to *Pr* such that the firing rate was kept constant across the conditions.

Previous theoretical studies have shown that including STD synapses in a homogenous recurrent network can result in PSs as transient network instability for a certain range of parameters (Tsodyks et al., 2000; Loebel and Tsodyks, 2002). In our network we add noise to the excitatory units such that the PSs are triggered by current fluctuations (Figure 2A). Noisy homogenous networks with STD exhibit two dynamical regimes; asynchronous activity and global synchronous activity. During the state of asynchronous activity the units fluctuate around their mean firing rate while during synchronous activity spontaneous synchronized PSs can be observed. A new, intermediate dynamical regime exists in a clustered network for which each unit can emit PS as a result of synaptic input fluctuation, yet there is no complete synchronization between the units (Figure 2A). The synchronous activity can be controlled such that the network shifts gradually from state of asynchronous activity to a global synchronization (Figure 2B), consequently there is a continuum range of synchronization as was suggested experimentally (Harris and Thiele, 2011).

Transmission of synchronized changes in pre-synaptic activity via depressing synapses strongly depends on the release probability; in particular, post-synaptic response becomes more transient as *Pr* increases (Tsodyks and Markram, 1997). We therefore conjectured that *Pr* is the natural parameter for controlling PSs in recurrent networks. Our simulations confirm this prediction (Figure 2A). Small fluctuation in external inputs cannot be enhanced by recurrent connections with low *Pr*, and increasing *Pr* above a certain value enables units to produce PSs. If *Pr* is not too large PS in one unit cannot initiate a PS in another unit, therefore there is no synchronization between the clusters. Increasing *Pr* further results in higher effective synaptic connections within and between the excitatory units, and in higher amplitude of PS, such that PS in one unit trigger PS in other units and the whole network synchronizes (Figure 2A).

While increasing the *Pr*, we decrease the average external current into the excitatory units in order to keep the firing rate constant, thus constraining the dynamics (Figure 2C).

Varying these two parameters simultaneously is biologically plausible. For example, acetylcholine (ACh), which is a neuromodulator that is involved in the regulation of transition between behavioral states (Steriade et al., 1993), both reduces the release probability in cortical pyramidal cells and depolarized the membrane potential (McCormick and Prince, 1986; Giocomo and Hasselmo, 2007).

### Optimal dynamical state

**Figure 3:**
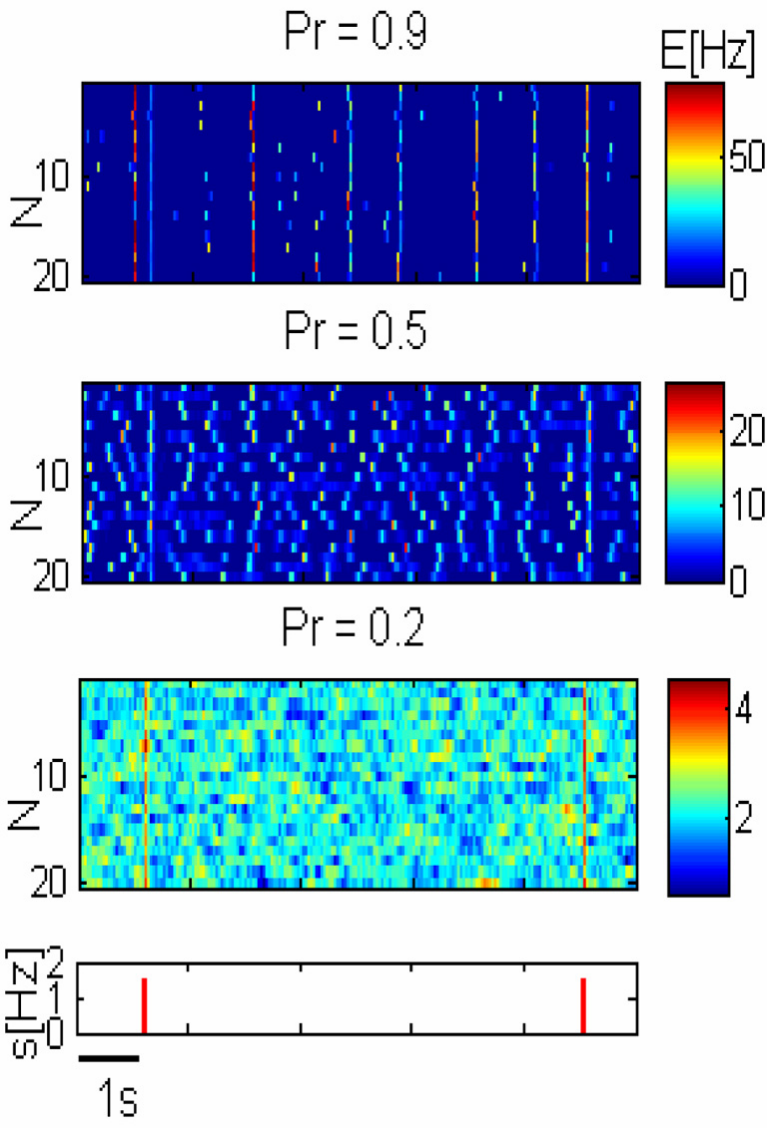
**Network response to pulse stimuli.** Three *upper panels*— response of three networks illustrated in Figure 2A to external inputs. *Lower panel*—inputs are shown as red pulses. Response amplitude grows with the release probability (*Pr*) but spontaneous global synchronization is observed with high *Pr*.

It was previously suggested that synchronization of neuronal activity is important for signal propagation in the cortex (Abeles, 1991). We examined the effect of synchronization in the form of PS on the flow of information between two networks representing two distinct areas in the brain. The first is the network described in the previous section which receives the sensory stimuli and the second is a readout population. The sensory input is taken to be an excitatory pulse that arrives at random times (See “Methods”). The response of the network to stimuli is shown in Figure 3.

**Figure 4:**
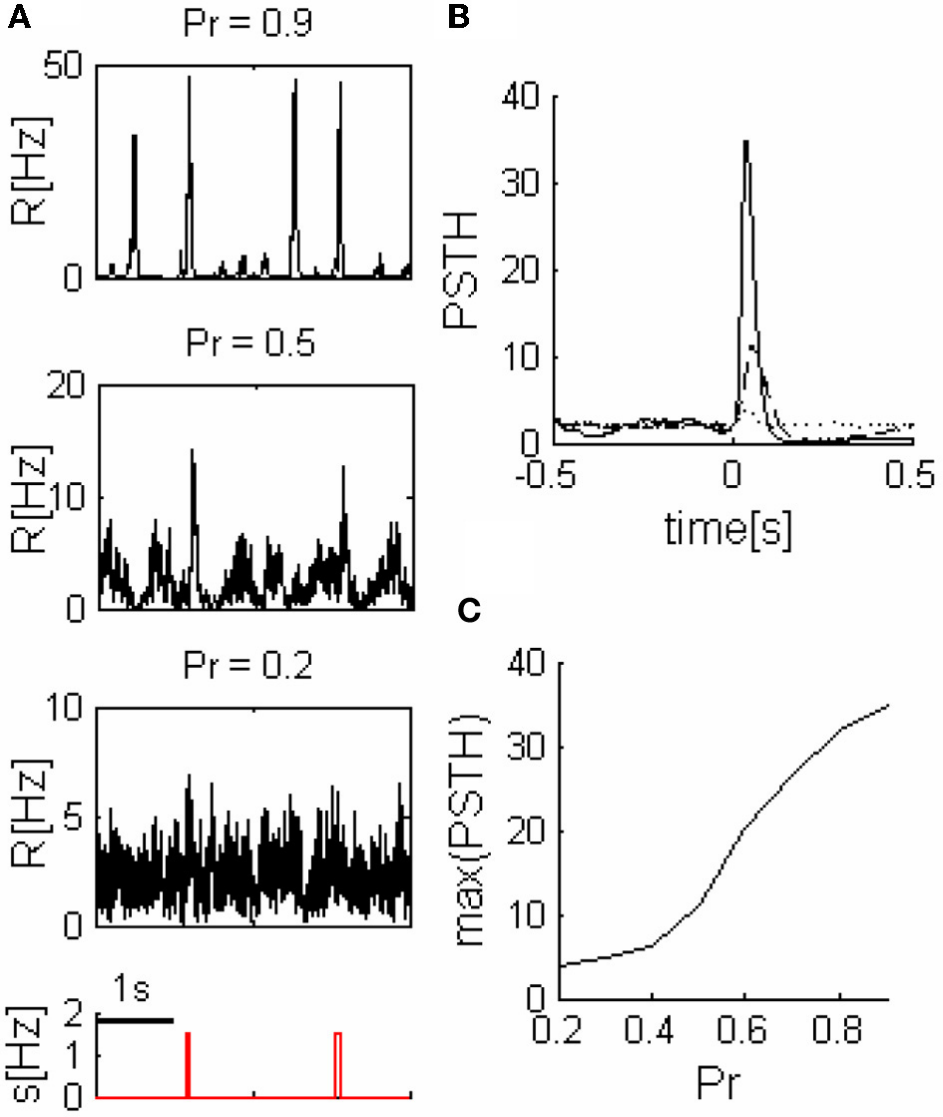
**Readout response to pulse stimuli. (A)** Readout response to external inputs (network parameters as in the three networks illustrated in Figure 2A). **(B)** Examples of readout PSTH, **----** *Pr* = 0.2, **––** *Pr* = 0.5, and **——** *Pr* = 0.9. **(C)** Readout response amplitude grows with the release probability (*Pr*). *N*_*n*_ = 5 in all of the panels.

In order to consider how the network can transmit sensory stimuli to higher cortical areas, we added a readout population (*R*) that receives spike trains from the network (Figure 1). We modeled spike trains emitted by excitatory neurons belonging to a certain unit by constructing Poisson process with the corresponding rate. Since the connectivity between different cortical areas is very sparse (Anderson et al., 1998; Douglas and Martin, 2007), we assume that the number of feed-forward neurons from each excitatory unit (*N*_*n*_) that are connected to the readout population is small. Activity of the readout in response to stimuli is plotted in Figure 4.

We quantified readout performance by defining events as peaks of readout activity that cross a threshold and calculated the ratio (TPR) between the number of events during stimulus (true positive events-*TP*) and the sum of total number of events and *FNs* (See “Methods”). The *TPR* is a function of the threshold, thus we characterize the performance by the maximum of the *TPR* (*TPR*_max_), (we assume a readout can learn the optimum threshold, therefore, it is not a parameter of our model).

**Figure 5:**
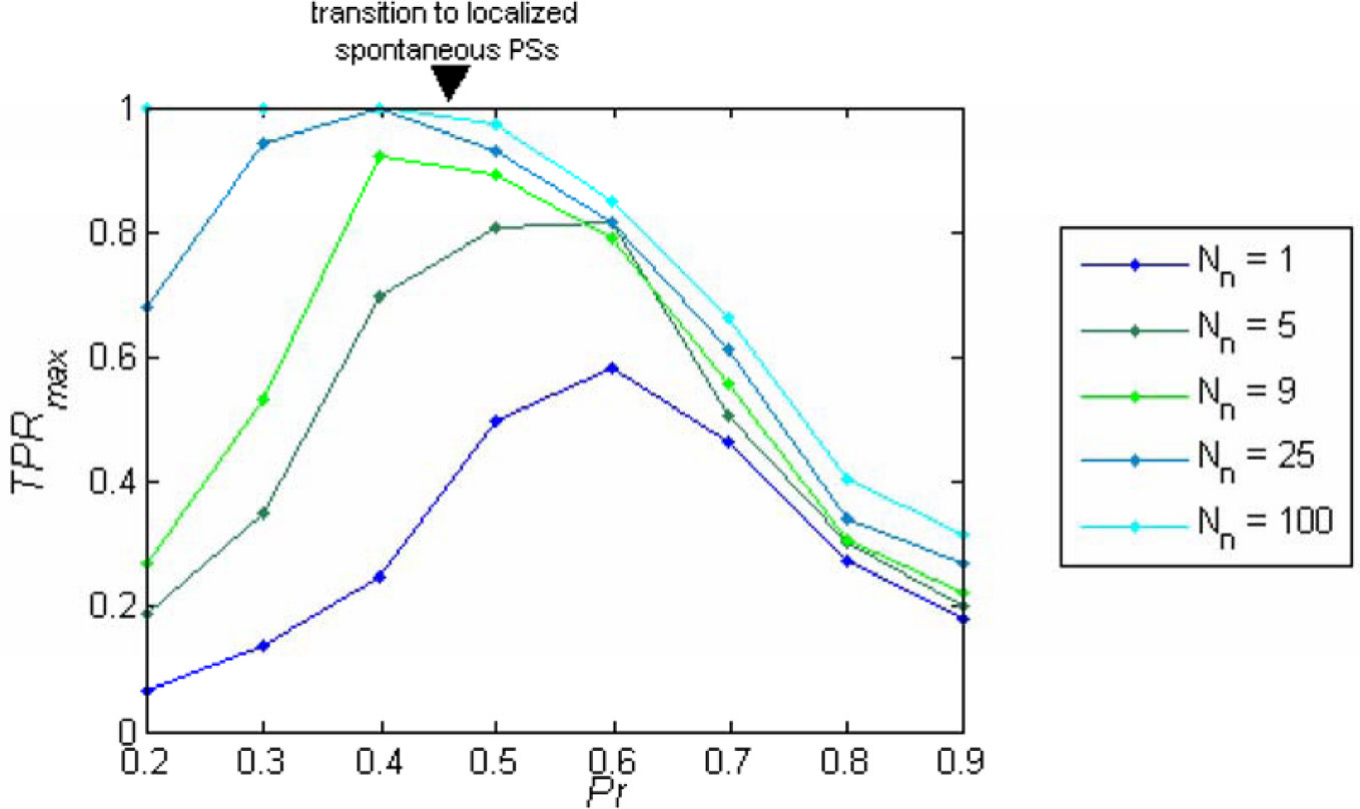
**Readout performance as a function of the release probability.** *TPR*_max_ is plotted as a function of the release probability (*Pr*) separately for each number of projecting neurons from each cluster (*N*_*n*_). The optimum *TPR*_max_ depends on *N*_*n*_; for *N*_*n*_ < 9 the maximum appears in the regime of localized spontaneous PSs, while with higher *N*_*n*_, the maximum shifts to the regime of asynchronous activity. With dense connectivity, the performance becomes a monotonically decreasing function of *Pr*.

The behavior of *TPR*_max_ as a function of *Pr* depends on the sparseness of the readout connections (*N*_*n*_). While for larger values of *N*_*n*_ the performance as a function of the release probability *TPR*_max_*(Pr)* is a monotonically decreasing function of *Pr* (Figure 5), for small enough values of *N*_*n*_ (sparse connectivity) this function exhibits a peak at a certain value of *Pr* (Figure 5) which corresponds to the regime of intermediate synchronization in the network.

### What determines the optimal state?

**Figure 6:**
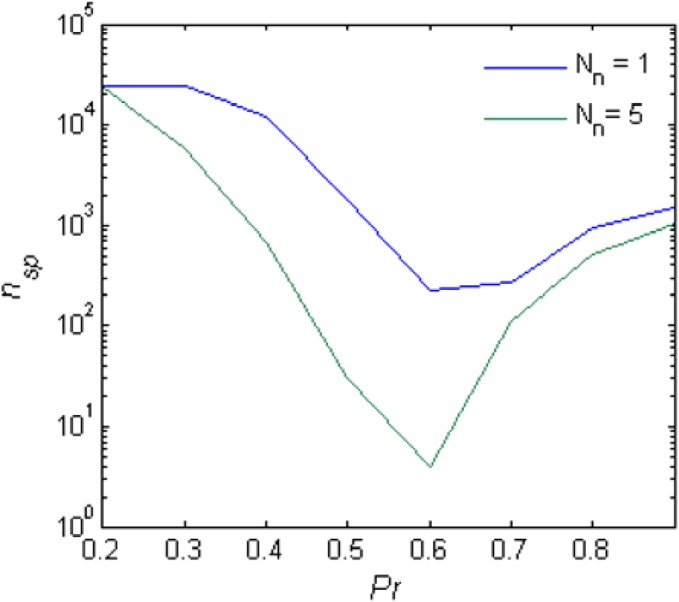
**Spontaneous peaks in activity.** The number of spontaneous events that cross the maximal PSTH value (*n*_sp_) as a function of the *Pr*.

For small *Pr*, the amplitude of network response to external stimulus is low, (Figure 3). This implies that for each input, only a small and highly variable fraction of neurons will emit a spike. Because the sampling of neurons by the readout is sparse, its response will be unreliable (within the noise level, Figure 4A). Consequently, the number of spontaneous events in the readout that cross the maximal PSTH value (*n*_sp_) is high. With large *Pr* values, the magnitude of networks spontaneous events (PSs) are in the same range as the responses (Figure 3), *hence*, *n*_sp_ is also high, independently on the number of sampled neurons (Figure 6). For the intermediate level of *Pr* the PSs synchronized across clusters as a result of stimuli while there is no spontaneous PSs synchronization, therefore, *n*_sp_ is small. Low values of *n*_sp_ enable a good performance for an appropriately chosen threshold (Figure 4). With sparse connectivity, *n*_sp_ has minimal value in the regime of local PSs (Figure 6) that corresponds to maximal *TPR*_max_(Figure 5).

In summary, Network and readout responses to stimuli are growing with *Pr* (Figures 3, 4), but spontaneous synchronized events occur for networks with high *Pr* (Figure 3), which can be erroneously recognized by the readout as inputs (false alarms). Hence, the fully synchronized regime is not beneficial for flow of sensory information. In the regime of intermediate synchronization, network response to stimuli is stronger than in the asynchronous state, while there are less FP events (Figure 3). Stronger network response results in higher probability of each neuron to fire action potentials in response to stimuli and consequently the flow of information in the sparsely connected cortex is more reliable. This advantage disappears when the sampling size (*N*_*n*_) increases and thus the optimal *Pr* shifts to smaller values.

### Integrate and fire networks

**Figure 7:**
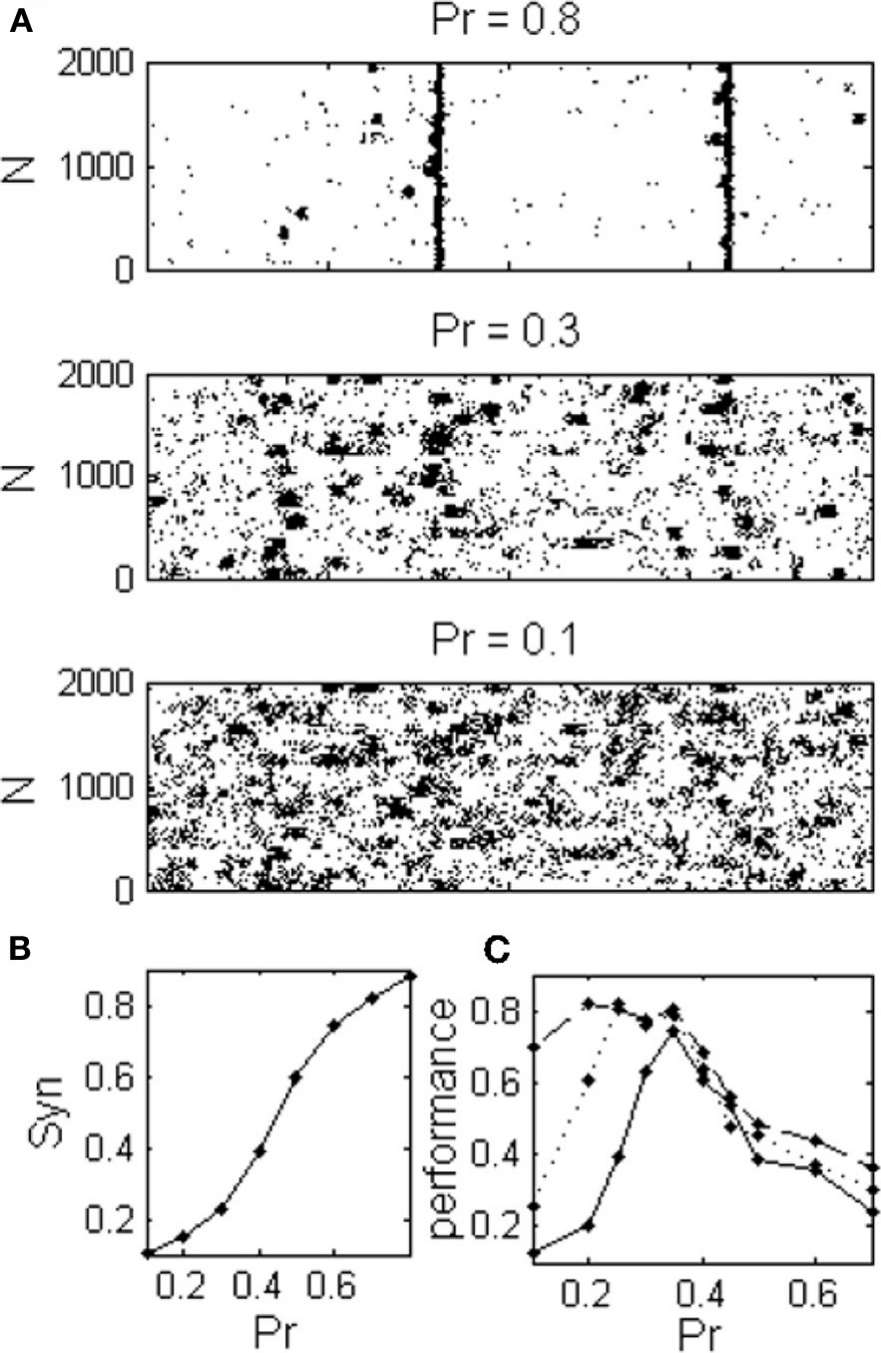
**Integrate and Fire network exhibits the same dynamical regimes as in the rate model. (A)** Raster plot shows spontaneous activities of excitatory neurons during three different dynamical regimes corresponding to different values of the release probability; asynchronous, localized synchronization, and global synchronization. **(B)** Network exhibits a continuum range of synchronization **(C)** The optimal regime for information transfer depends on the connectivity to the readout population as in the rate model, (*N*_*n*_: **———** one connection for every third cluster, **----** *N*_*n*_ = 1 and **––** *N*_*n*_ = 9 connections from each cluster).

In order to verify that the results obtained with the rate model remain valid in a more realistic model, we simulated networks of I&F spiking neurons (See “Methods”). The three dynamical regimes: asynchronous dynamics, local synchronization and global synchronization, were also observed in I&F networks (Figures 7A,B). The optimal regime for information flow between sparsely connected networks is again the intermediate dynamical regime (Figure 7C). Only with denser sampling, (more than *N*_*n*_ = 9, approximately 10% of the network neurons), the performance becomes a monotonically decreasing function of *Pr*. The problem of sparse connectivity will be even more acute if we consider a more realistic case of noisy readout (not shown).

## Discussion

We proposed a mechanism by which the synchronization of network activity can be generated and regulated. As previous studies showed, homogenous networks with STD exhibit two dynamical regimes: asynchronous and globally synchronized activity in the form of PS. In this study we modeled a cortical column as a clustered recurrent network. We report that networks with clustered architecture possess a new dynamical regime in which groups of neurons (clusters) emit PSs that are not fully synchronized between the groups. This regime is further divided into a continuum of states with gradually changing levels of global synchronization that can be controlled by network parameters, as was suggested by experiments (Harris and Thiele, 2011). We showed that reduction in release probability results in de-synchronization of neuronal activity.

Our proposed mechanism for transition between dynamical states may be implemented in the cortex by neuromodulators such as ACh (Goard and Dan, 2009). ACh can regulate STD in the cortex by its effect on the probability of neurotransmitter release (Tsodyks and Markram, 1997), therefore controlling the synchronization that is generated in the cortex by the mechanism proposed in this study. Indeed, ACh is involved in the regulation of transition between behavioral states, in particular, its secretion increases in the cortex when the animal is in the alert state (Perry et al., 1999; Giocomo and Hasselmo, 2007).

Previous work demonstrated variable dynamic state, with different synchrony levels, in recordings of cortical activity in the urethane anesthetized rats. The data was fitted to a dynamical system such that every state characterized by different set of parameters. They showed that the synchronized cortical states can be modeled as self-exciting system while the most desynchronized state is better approximated with a linear dynamics (Curto et al., 2009). Here we used biologically plausible dynamics in which cortical state is regulated via the release probability that controls the non-linearity of the dynamics; therefore shift it from non-linear in the synchronized state to approximately linear in the desynchronized state.

It has been suggested that synchronization of neuronal activity is beneficial for information flow in the sparsely connected cortex (Singer, 1993). In our model, during PSs the neurons have higher probability to fire as a result of the synchronous activity; therefore it may be a mechanism by which primary cortical populations overcome their sparse connectivity to remote areas in the brain in order to transfer further the sensory information. However, it has some disadvantages: large activity during PS synchronizes the clusters and increases the occurrence of false detections. Depletion in the synaptic resources, as a result of synchronous PSs, prevents column responses to an incoming stimulus that appears in a short time interval after strong synchronous network event. Our simulation results imply that the best regime for information transfer would be when PSs occur within clusters but there is no synchronization across clusters.

Further suggestions can be made concerning the possible role of clustered architecture. Inspired by the patchy long distance connection in primary sensory areas (Gilbert and Wiesel, 1989; Amir et al., 1993) and the binding theory (Singer, 1993), we can hypothesize that different clusters which belong to the same column, responding to the same feature such as orientation, tone etc, are involved in the coding of complex stimuli (such as combination of features) by synchronized their activity with part of other columns clusters. Then, synchronous PSs increase the noise correlation between the clusters which may encode different complex stimuli. The regime of asynchronous PSs within groups will be also more beneficial in encoding these various features combinations. Moreover, it may be interesting to examine what can be the functional role of the other dynamical regimes. For example, it has been suggested that synchronous activity is important for plasticity (Singer, 1993; Sejnowski and Destexhe, 2000), therefore it may be interesting to examine what could be the effect of PS generation and synchronization on memory consolidation.

In summary, our results illustrate that the cortical networks can exhibit very different activity regimes most suitable for a particular behavioral state. In the case of sensory processing that we considered in this study, choosing the right regime is beneficial for signaling the sensory inputs to higher brain areas. We believe, however, that all the cortical circuits are capable of performing several functions, and have to be tuned to the particular behavior depending on the computational demands of the area. The mechanisms by which the brain can achieve this tuning are probably diverse and should be a subject of further theoretical studies.

### Conflict of interest statement

The authors declare that the research was conducted in the absence of any commercial or financial relationships that could be construed as a potential conflict of interest.
